# The battle for mental well-being in Ukraine: mental health crisis and economic aspects of mental health services in wartime

**DOI:** 10.1186/s13033-023-00598-3

**Published:** 2023-09-25

**Authors:** Violetta Seleznova, Irina Pinchuk, Inna Feldman, Volodymyr Virchenko, Bo Wang, Norbert Skokauskas

**Affiliations:** 1 Faculty of Economics, Taras Shevchenko National University of Kyiv, Kyiv, Ukraine; 2 Institute of Psychiatry, Taras Shevchenko National University of Kyiv, Kyiv, Ukraine; 3 Department of Public Health and Caring Sciences, Social Medicine (CHAP), Uppsala University, Uppsala, Sweden; 4 Norwegian Centre for E-health Research, University Hospital of North Norway, Tromsø, Norway; 5 Department of Mental Health, Regional Centre for Child and Youth Mental Health and Child Welfare (RKBU Central Norway, Norwegian University of Science and Technology (NTNU), Trondheim, Norway

**Keywords:** Russian-Ukrainian war, Mental health crisis, Mental health care system, Mental well-being, Mental health economics, Capacity building

## Abstract

The ongoing war in Ukraine is having profound impacts on both the local and global economy, as well as the infrastructure and overall well-being of the people. The prolonged duration of the conflict, coupled with its many related consequences such as total uncertainty, unfavorable economic conditions, and a distressing media backdrop, have a lasting impact on the mental health of the population. The ongoing war in Ukraine has exposed weaknesses in the national mental health care system and underscored the importance of mental health economics. To prevent further mental health problems, it is crucial to develop a comprehensive set of measures aimed at strengthening the capacity of the mental health care system in Ukraine. Currently, Ukraine’s mental health care system suffers from a lack of financial and human resources, which hinders its ability to provide adequate support to those in need. To address this issue, joint efforts between Ukrainian mental health stakeholders and the international governmental and non-governmental organizations are needed to provide support and capacity building for mental health services in Ukraine.

## Introduction

War has both immediate and long-term public health consequences: people can be killed or injured from violence itself, or can develop health problems stemming from the traumatic experience of war and the scarcity of access to adequate health care. War can affect people at any life stage — from infancy and early childhood to adulthood — for long periods of time, but children are probably most profoundly affected by war, given the utmost importance of the early years in a child’s life [1, 2].

The large-scale invasion of Ukraine by the Russian Federation began on February 24, 2022. This phase was preceded by an eight-year lower-intensity armed conflict in Eastern Ukraine. The war has had devastating effects on the health and well-being of the Ukrainian nation, and has led to a rapid escalation of a mental health crisis [1]. Ukrainians have been exposed to a range of traumatic events, such as witnessing or experiencing war traumas, and the death of loved ones, leading to high rates of post-traumatic stress disorder and depression [3, 4].

At time time of writing this paper (spring 2023), about 14% of Ukrainian lands remain under occupation [5]. Many homes were destroyed or damaged [6]. Tens of millions of Ukrainians were displaced within the country and abroad, causing one the largest migration crisis of the century. More than 5 million Ukrainian refugees have a temporary protection in Europe [7]. Approximately 17% of Ukrainians have lost contact with friends and relatives and do not know about their destiny [8]. Families are torn apart: men cannot leave the country, and it is difficult to evacuate the elderly from front-line settlements to less dangerous ones. At least 19,000 children were deported to Russia [9].

Those who stayed in Ukraine, in addition to physical danger, face financial challenges on a daily basis. According to one of the latest surveys, 78% of Ukrainians declared a decrease in income, and about a third lost their jobs due to the outbreak of war [10]. Meanwhile, in 2022, consumer prices in Ukraine increased by 20% compared to 2021 [11]. Missile attacks on energy infrastructure have left many Ukrainian households living and working amid long blackouts, disrupting the usual rhythm of life and reducing the productivity.

The direct and indirect losses caused by the military aggression may affect the mental health of entire generations of Ukrainians. The experience of the war traumatizes Ukrainian society, while infrastructural destruction and economic decline complicate the access to quality mental health services. The impact of the war on mental health, as well as the needs and accessibility of mental health serices, espcially for internal displaced persons should be thoroughly studied in order to develop effective solutions for strengthening the Ukrainian mental health care system. In addition, the war in Ukraine clearly demonstrates the weakest aspects of the national mental health care: it has increased the need for mental health care while simultaneously reducing the capacity of the national health care system.

## The state of the mental health service before the full-scale invasion

Ukraine has inherited the Soviet mental health care system, which is characterized by an overconcentration of psychiatric institutions with very limited community mental health services [12], as well as strong mental illness stigma [13]. Prior to the full-scale war, appoximately 30% of Ukrainians suffered from a mental health disorders throughout their lives [14]. In 2019, the prevalence of depressive disorders in Ukraine exceeded the average for the European Union — 5,2% versus 4,6% accordingly [15], and there were 22 deaths by suicide per 100,000 in Ukraine compared to 11 ones in the European Union [16].

The war started in 2014 in the Eastern Ukraine exposed problems in the mental health care. Over an 8-year period, 4,400 Ukrainian forces and 3,404 civilians were killed, about 14,000 Ukrainian forces and approximately 8,000 civilians were wounded [17]. There were from 700 thousand [18] to 1.5 million [19] internally displaced civilians. Ukrainian adolescents who were victims of violence were more than 4 times as likely to develop post-traumatic stress disorder [20].

A third of internally displaced people had symptoms of post-traumatic stress disorder, 22% had depression, 18% had anxiety, and 30% had severe psychological distress [21, 22]. Despite the significance of the problem caused by the military conflict in 2014–2021, the state response to mental health challenges was insufficient. Only 10% of internally displaced people with mental disorders received professional help [23]. Only five years after the start of the war, a 24-hour national helpline for veterans was launched [24]. In 2018, the Ministry of Veterans Affairs was established, and two years later, the Ministry for Temporarily Occupied Territories and internally displaced people (later renamed the Ministry of Reintegration) began its work. Their activities were partly related to the mental healthcare of groups affected by the war. According to survey, funding for national mental health care facilities was reduced by 50·25% for the April – December 2020 compared with the same period in 2019: it was equivalent to the loss of more than 3 thousand full-time positions, approximately 2.5 of which were health professionals (doctors, nurses, paramedics, psychologists and social workers) [25].

## Impact of a full-scale invasion on mental health services in Ukraine

The large-scale invasion of Ukraine by the Russia Federation began on February 24, 2022. In 2022, there were 707 attacks on Ukrainian health care facilities, 218 hospitals and clinics were damaged or destroyed, 181 attacks on other medical infrastructure facilities (pharmacies, dentistry) were documented [26]. The damage caused by the aggression to the healthcare system of Ukraine was 26 billion dollars [27]. During the year, there were 86 known attacks on health care workers, with 62 health workers killed [26]. Many medical workers were injured, threatened and harassed. Some were taken hostage, illegally imprisoned or forced to work under the occupation [26].

The greatest damage to the medical infrastructure was recorded in the eastern regions of Ukraine [26]. During the three months of full-scale aggression, 80% of the facilities for the provision of medical services in Mariupol were destroyed or damaged [28]. The only psychiatric hospital in the city was destroyed [29]. Attacks were carried out on psychiatric hospitals in Kramatorsk [30], Kherson region [31], psycho-neurological boarding houses in Kyiv [32], Kharkiv [33], and Sumy [34] regions. While working on this comment, on Easter night, an air raid was carried out on another psycho-neurological boarding house [35]. The mental health services structure suffered from a lack of personnel due to the injuries and evacuation of medical workers [36].

During the first 8 months of a full-scale invasion, 650,000 Ukrainians received professional help from psychologists and psychiatrists [37]. One study reports that more than 80% of the Ukrainians have never consulted a psychologist or psychotherapist, although at least a third have recently experienced irritability, poor sleep, bad mood, tension and anxiety (2,100 respondents) [38]. The most frequently mentioned obstacle in accessing mental health care services has been cost [39], but also availability and long lines at local pharmacies [40]. In the first month of full-scale invasion, more than 2,000 people on opioid-substitution therapy were at risk of treatment interruption [41]. These problems are highlightling the siginificant impact of war on the accessiblity of health care services. WHO Country Office in Ukraine and the Ministry of Health of Ukraine predict between 10 and more than 15 million Ukrainians who will need professional psychological assistance as a result of hostilities [40, 42]. Health providers have been also experiencing mental health problems: 40% of the “Stop Panic” hotline service staff, who were a key source of psychological support for the population in the first months of the war, had symptoms of depression and anxiety (as of March 18–26, 2022) [43].

Based on the few available studies, the Ukrainian mental health care system lacks financial resources [21, 25, 44–46], workforce capacity and accessability of services [21, 22, 25, 36, 45–47]. While insufficient funding for mental health services is common, in Ukraine this situation is dramatically worsened by the war and relicts from Soviet era including overfocus on inpatient care, high out-of-pocket payments and low staff wages leading to workforce shortages [21, 46].

## Conclusions

The war has increased the demand for quality mental health assistance and demonstrates the vulnerabilities of the current mental health system in Ukraine.

A careful planning process will need to start with an analysis of the gap between available resources and the need for expanded mental health services in Ukraine. Lack of studies that adequately describe the economic dimensions in the mental health care system of Ukraine limits the development of effective measures to overcome the existing problems in this health care sector. Future work is necessary to carry out a fundamental and comprehensive review of mental health services in Ukraine in order to design a mental health system that can serve the needs of the population effectively and efficiently. Partnership with international organization would be of great benefit since they could bring much needed expertise and resources.

## Data Availability

All of data and materials are freely available to use for non-commercial purposes, no permissions are required.
